# Actinic Skin Damage and Mortality - the First National Health and Nutrition Examination Survey Epidemiologic Follow-up Study

**DOI:** 10.1371/journal.pone.0019907

**Published:** 2011-05-13

**Authors:** Wei He, Fei Zhu, Xiaoguang Ma, Xinyu Zhao, Min Zheng, Zhao Chen, Steven B. Heymsfield, Shankuan Zhu

**Affiliations:** 1 Injury Control Research Center, Obesity and Body Composition Research Center, School of Public Health, Zhejiang University, Hangzhou, China; 2 Injury Research Center, Medical College of Wisconsin, Milwaukee, Wisconsin, United States of America; 3 Department of Epidemiology and Biostatistics, Arnold School of Public Health, University of South Carolina, Columbia, South Carolina, United States of America; 4 Department of Dermatology, Second Affiliated Hospital, Zhejiang University, Hangzhou, China; 5 Division of Epidemiology and Biostatistics, Mel and Enid Zuckerman College of Public Health, University of Arizona, Tucson, Arizona, United States of America; 6 Pennington Biomedical Research Center, Baton Rouge, Louisiana, United States of America; Women's College Research Institute, University of Toronto, Canada

## Abstract

**Background:**

Exposure to sunlight may decrease the risk of several diseases through the synthesis of vitamin D, whereas solar radiation is the main cause of some skin and eye diseases. However, to the best of our knowledge, the association of sun-induced skin damage with mortality remains unknown.

**Methodology/Principal Findings:**

Subjects were 8472 white participants aged 25–74 years in the First National Health and Nutrition Examination Survey Epidemiologic Follow-up Study. Cardiovascular disease mortality, cancer mortality, and all-cause mortality were obtained by either a death certificate or a proxy interview, or both. Actinic skin damage was examined and recorded by the presence and severity (absent, minimal, moderate, or severe) of overall actinic skin damage and its components (i.e., fine telangiectasia, solar elastosis, and actinic keratoses). Cox regression and Kaplan-Meier methods were applied to explore the associations. A total of 672 cancer deaths, 1500 cardiovascular disease deaths, and 2969 deaths from all causes were documented through the follow-up between 1971 and 1992. After controlling for potential confounding variables, severe overall actinic skin damage was associated with a 45% higher risk for all-cause mortality (95% CI: 1.22, 1.72; *P*<0.001), moderate overall skin damage with a 20% higher risk (95% CI: 1.08., 1.32; *P*<0.001), and minimal overall skin damage with no significant mortality difference, when compared to those with no skin damage. Similar results were obtained for all-cause mortality with fine telangiectasia, solar elastosis, and actinic keratoses. The results were similar for cancer and cardiovascular disease mortality.

**Conclusions:**

The present study gives an indication of an association of actinic skin damage with cardiovascular disease, cancer and all-cause mortality in white subjects. Given the lack of support in the scientific literature and potential unmeasured confounding factors, this finding should be interpreted with caution. More independent studies are needed before any practical recommendations can be made.

## Introduction

Exposure to sunlight can provide people with a sense of wellbeing and a tanned body is viewed as a sign of good health. [1], [2] Humans get vitamin D from exposure to sunlight, which may play a role not only in the maintenance of healthy bones, but also in reducing the risk of some illnesses, including autoimmune, infectious, and cardiovascular diseases, and colon, prostate, and breast cancers.[3] In addition, artificial ultraviolet (UV) radiation is used to treat several diseases under medical supervision, including rickets, psoriasis and eczema. [4]

However, overexposure to the sun also carries potential risks to human health. UV radiation is a known carcinogen and excessive exposure increases the risk of lip, skin basal cell and squamous cell carcinomas and cutaneous melanoma, particularly in fair-skinned population. [5] There is also evidence that solar UV radiation increases the risk of several eye diseases, including cortical cataract, some conjunctive neoplasms, and possibly ocular melanoma. [5]

Chronic repeated sunlight exposure can also induce degenerative changes in skin cells, fibrous tissue, and blood vessels, leading to visible signs of actinic skin damage, [4] which is partially characterized by telangiectasia, solar elastosis, and actinic keratoses. [6], [7], [8], [9] Epidemiologic studies have found that people with accelerated aging looking have an increased mortality risk, [10], [11] and the skin damage caused by overexposure to sunlight is an important environmental factor for increased facial “aging”. [12] However, we are not aware of any study designed to investigate the relation between sun-induced skin damage and mortality.

The present study aims to examine the associations of actinic skin damage with cardiovascular disease mortality, cancer mortality, and all-cause mortality in whites based on the data from the First National Health and Nutrition Examination Survey (NHANES I) Epidemiologic Follow-up Study (NHEFS), which is characterized by a large sample size and an average of a 16-year follow-up.

## Methods

### Ethics statement

The primary NHANES I and NHEFS were approved by the National Center for Health Statistics (NCHS) Institutional Review Board.[13] The present study was approved by the Ethics Committee of Zhejiang University School of Public Health.

### Participants

The First National Health and Nutrition Examination Survey (NHANES I) was conducted between 1971 and 1975 by the National Center for Health Statistics based on a probability sample of the civilian noninstitutionalized population of the United States. [14], [15], [16] The NHANES I Epidemiologic Follow-up Study (NHEFS) is a longitudinal follow-up of participants in NHANES I who were aged 25 to 74 years at the time of survey. [17] Detailed information on NHANES I and NHEFS are provided elsewhere. [14], [15], [16], [17]

Of the 12, 053 white subjects followed in the NHEFS, we excluded 2760 NHANES I Augmentation Survey participants who were not administered a dermatologic examination[16]; 83 subjects who were pregnant at the time of the baseline examination, and 487 subjects whose demographic, socioeconomic, or other covariates were missing. Among the remaining participants, 251 were lost during the follow-up period, leaving 8472 participants (men, 40.5%; women, 59.5%) who contributed 139,802 person-years of follow-up to the final analyses.

### Outcome definitions

Based on interviews, health care facility medical records, and death certificates, NHEFS follow-up data were collected in 4 waves: 1982 to 1984, 1986, 1987, and 1992. [17] Death was ascertained by either a death certificate or a proxy interview, or both, and the underlying cause of death was coded according to the International Classification of Diseases, Ninth Revision (ICD-9). Deaths were analyzed for all causes, cancer (ICD-9 codes 140–208, e.g., lung cancer, colon cancer, breast cancer), and cardiovascular disease (ICD-9 codes 390–448, e.g., ischemic heart disease, cerebrovascular diseases, hypertension).

### Actinic skin damage assessment

A complete clinical dermatologic examination of the skin was undertaken in NHANES I to evaluate variations in texture and color, certain manifestations of aging, and all pathologic changes. The dermatologic examinations were performed by a total of 101 dermatologists during the baseline survey. All dermatologists were trained for the dermatologic examination protocol before the commencement of the survey examinations at each location. After examining the skin covering the entire body, the dermatologist recorded an estimate of the presence and severity of overall actinic skin damage and its components, including fine telangiectasia, solar elastosis (senile elastosis), and actinic keratoses. All of these measures were graded as being either absent, minimal, moderate, or severe. [18]

### Covariate definitions

Several potential confounding factors derived from the baseline interview and physical examinations were included in the regression models based on findings from previously published studies. [18], [19], [20] These included age, gender, educational attainment (<12^th^, 12^th^, >12^th^ grade); family income (<$3000, $3000–$7000, $7000–$10000, $10000–$15000, ≥$15000); combined measure of occupational and recreational physical activity (low, medium, high); body mass index (underweight, normal, overweight, obese); vitamin supplement use (regular or irregular use, no); place of residence (rural, urban); region of residence (Northeast, Midwest, South, West); season of the year when the examination was taken (spring, summer, autumn, winter); eye color as a surrogate of skin type [21] (dark brown, light brown, gray-green-hazel, dark blue, light blue, other); frequency of alcohol consumption (less than once a month or never, once a month to several times a week, almost daily or daily). Menopause was defined by the answer “yes” for the question, “have the cycles entirely stopped?”. Occupation was coded according to the 1970 census of population Alphabetical Index of Industries and Occupations [22]: professional, manager, sales worker, clerical and kindred workers, craftsmen and kindred workers, operatives, laborer, farmer, service works, and unemployment variables for retired, keeping house, and others (disabled, student, looking for work, missing). Smoking was coded as current, past, or never. Past smokers were defined as those who reported that they had smoked ≥100 cigarettes during their lifetimes but did not currently smoke cigarettes. Retrospective smoking data collected during the follow-up were used for persons with missing baseline information. Retrospective information on smoking agreed reasonably well with data collected in NHANES I. [23] Baseline diseases were defined as self-reported history of having cardiovascular diseases, cancer, diabetes, hypertension, ulcer, kidney diseases or hepatitis.

### Statistical methods

Characteristics of the participants at baseline were presented as the mean or percentage stratified by severity of overall actinic skin damage. The level of statistical significance of differences were examined by using ANOVA (continuous variables) and the χ^2^ test (categorical variables), with Bonferroni adjustment for multiple comparisons.

Cox proportional hazards regression models [24] were used separately to examine the associations of overall actinic skin damage and its components with all cause, cancer, and cardiovascular disease (CVD) mortality. [25] Age was used as the time scale in all time-to-event analyses, and all analyses were stratified by birth cohort using 10-year interval to control for calendar period and cohort effect. [26] Trend tests for the adjusted hazard ratios (HRs) were performed by assigning the integers of 0, 1, 2, 3 to the degrees of severity of actinic skin damage and its components, and treating the resulting score variables as continuous in the models. The Schoenfeld residual [27] test was used to check the proportional hazards assumption. In addition, we also performed the Cox regression model with time-on-study as the time-scale and baseline age as a covariate.

To assess the effect of the complex survey design on the results, Cox proportional hazard regression analyses were performed to incorporate the sample weights, the stratification and clustering in the analysis. The weighted and unweighted models yielded consistent results. Therefore, only unweighted results were presented here. To explore whether the results will be affected by skin cancer, we also performed the analyses excluding those who died from skin cancer during the follow-up period (n = 8) in all three models. Subgroup analyses by age (25–44, 45–65, 65–74 years old) were conducted to assess the robustness of our findings. Cox regressions were also performed excluding subjects with cardiovascular disease, diabetes, or cancer at baseline, and were repeated when including pregnant women. All Cox models were adjusted for the above-mentioned potential confounding factors. In addition, men and women were combined in the regression analysis as no interactions were detected between gender and actinic skin damage.

Cumulative mortality curves were plotted according to overall actinic skin damage severity using the Kaplan-Meier method. [28] The assessment of cumulative mortality rates was limited to subjects who were 40–85 years of age during the follow-up period. Because the calculation of cumulative mortality rates is sensitive to small sample sizes, moderate and severe actinic skin damage were combined into one group. Pairwise comparisons of absent, minimal, and moderate and severe overall actinic skin damage were performed using a two-sided log-rank test [29] for all-cause (n = 8431), cancer (n = 6154) and cardiovascular disease (n = 6981) mortality, respectively. Bonferroni corrections were used to control for type I error, and a two-sided P value of 0.017 was used for each comparison. All analyses were performed using Stata software (version 11.0 for Windows; Stata Corporation, College Station, TX).

## Results

The baseline characteristics of participants by overall actinic skin damage are presented in **Table 1**. Compared with women, men were more likely to have overall actinic skin damage. The severity of overall actinic skin damage increased with age and decreased with family income, education attainment, and vitamin supplement use. Subjects who lived in the South, rural or with light color eyes were more likely to suffer from severe overall actinic skin damage.

**Table 1:** Baseline characteristics of 8472 NHEFS participants according to actinic skin damage*.

	Actinic skin damage			
Variable	Absent(4419)	Minimal(2125)	Moderate(1710)	Severe(218)
Age(years), mean(SD)	43.4(14.8)	54.7(14.1)^a^	60.4(11.6)b,d	64.6(8.5)c,e,f
Body mass index(Kg/m^2^), mean(SD)	25.2(5.1)	25.8(4.8)^a^	25.7(4.5)^b^	25.4(4.7)
Men, No. (%)	1311(29.7)	878(41.3)^a^	1083(63.3)b,d	161(73.9)c,e,f
High physical activity, No. (%)†	604(13.7)	294(13.8)	260(15.2)	25(11.5)
Current cigarette Smoke, No. (%)	1212(27.4)	452(21.3)^a^	367(21.5)^b^	45(20.6)
Regular alcohol consumption, No. (%)‡	474(10.7)	270(12.7)	294(17.2)b,d	24(11.0)
More than a high school education, No.(%)	1163(26.3)	436(20.5)^a^	290(17.0)b,d	31(14.2)^c^
Family income≥$10000, No. (%)	2057(46.6)	830(39.1)^a^	557(32.6)b,d	51(23.4)c,e,f
Vitamin supplements use, No. (%)§	1564(35.4)	746(35.1)	515(30.1)b,d	61(28.0)
Dark brown eyes, No. (%)	1184(26.8)	385(18.1)^a^	239(14.0)b,d	19(8.7)c,e
Laborer or farmer, No. (%)	102(2.3)	53(2.5)	51(3.0)	7(3.3)
Residence in rural, No. (%)	1729(39.1)	814(38.3)	741(43.3)b,d	117(53.7)c,e,f
Residence in the South region, No. (%)	1037(23.5)	506(23.8)	439(25.7)	101(46.3)c,e,f
Examination taken in summer, No. (%)	1192(27.0)	561(26.4)	413(24.2)	41(18.8)^c^
Baseline disease, No. (%)**	1745(39.5)	1023(48.1)^a^	966(56.5)b,d	151 (69.3) c,e,f

*Data were given as mean ± SD or numbers (%). NHEFS indicates the First National Health and Nutrition Examination Survey Epidemiologic Follow-up Study. *P* was corrected by Bonferroni adjustment in multiple comparisons (p/q, q = 6, *P*<0.008).

† High physical activity was defined as both get much exercise in recreational things and very active in usual day aside from recreation.

‡ Regular alcohol consumption: defined as drink everyday or just about everyday.

§ Vitamin supplements use was defined as the regular or irregular use of any type of vitamin supplement.

**Baseline diseases were defined as self-reported history of having cardiovascular diseases, cancer, diabetes, hypertension, ulcer, kidney diseases or hepatitis.

*^a^*Statistically significant difference between the minimal and absent group.

*^b^*Statistically significant difference between the moderate and absent group.

*^c^*Statistically significant difference between the severe and absent group.

*^d^*Statistically significant difference between the moderate and minimal group.

*^e^*Statistically significant difference between the severe and minimal group.

*^f^*Statistically significant difference between the severe and moderate group.

Eight thousand four hundred and seventy-two participants with 139, 802 person-years of follow-up between 1971 and 1992 were included in the analyses, and 672 cancer deaths, 1500 cardiovascular disease deaths, and 2969 deaths from all causes were documented among the subjects. HRs and 95% CIs for all-cause mortality by the severity of overall actinic skin damage and its components (i.e., fine telangiectasia, solar elastosis (senile elastosis), and actinic keratoses) were presented in **Table 2**. After adjusting for potential confounding factors, severe overall actinic skin damage was associated with a 45% higher all-cause mortality (95% CI: 1.22, 1.72; *P*<0.001), moderate overall skin damage with a 20% higher mortality (95% CI: 1.08, 1.32; *P*<0.001), and minimal overall actinic skin damage with no significant mortality difference (*p* = 0.752), when compared to having no skin damage. Similar results were obtained when exploring the associations of all-cause mortality with fine telangiectasia, solar elastosis, and actinic keratoses, respectively. Significant trends were found in all of the analyses. HRs and 95% CIs for cancer and cardiovascular disease mortality according to severity of overall actinic skin damage and its components are presented in **Tables 3** and **4**, respectively. The results were similar to those of all-cause mortality shown in Table 2.

**Table 2:** All-cause mortality hazard ratios according to actinic skin damage and its components in 8472 NHEFS participants*.

	Actinic skin damage and its components				*P* for trend
	Absent	Minimal	Moderate	Severe	
**Actinic skin damage**					
Person-years	78558	34371	24253	2621	
Number of subjects†	4419 (915)	2125 (856)	1710 (1026)	218 (172)	
HR (95% CI)					
Age- adjusted**	1.0	1.07(0.97, 1.18)	1.39^c^(1.27,1.52)	1.78^c^(1.51,2.10)	<0.01
Age-,gender-adjusted‡	1.0	1.01(0.92,1.11)	1.16^b^(1.06,1.28)	1.42^c^(1.20,1.69)	<0.001
Multivariate adjusted§	1.0	1.01(0.92,1.11)	1.20^c^(1.08,1.32)	1.45^c^(1.22,1.72)	<0.001
**Fine Telangiectasia**					
Person-years	92103	26151	18438	3111	
Number of subject†	5282 (1294)	1658 (741)	1288 (755)	244 (179)	
HR (95% CI)					
Age- adjusted**	1.0	1.06(0.97,1.16)	1.39^c^(1.27,1.52)	1.64^c^(1.40,1.92)	<0.001
Age-,gender-adjusted‡	1.0	0.99(0.90,1.08)	1.15^b^(1.04,1.26)	1.27^b^(1.08,1.49)	0.001
Multivariate adjusted§	1.0	0.98(0.89,1.07)	1.16^b^(1.05,1.28)	1.27^b^(1.08,1.50)	<0.001
**Solar Elastosis**					
Person-years	99096	20470	16831	3405	
Number of subject†	5663 (1352)	1312 (620)	1217 (775)	280 (222)	
HR (95% CI)					
Age- adjusted**	1.0	1.12^a^(1.02,1.23)	1.30^c^(1.18,1.42)	1.53^c^(1.32,1.76)	<0.001
Age-,gender-adjusted‡	1.0	1.06(0.97,1.17)	1.11^a^(1.02,1.22)	1.32^c^(1.14,1.53)	<0.001
Multivariate adjusted§	1.0	1.06(0.96,1.17)	1.15^b^(1.05,1.27)	1.35^c^(1.16,1.58)	<0.001
**Actinic Keratoses**					
Person-years	119640	12948	6047	1168	
Number of subject†	7004 (2012)	918 (564)	449 (308)	101 (85)	
HR (95% CI)					
Age- adjusted**	1.0	1.23^c^(1.12,1.35)	1.36^c^(1.21,1.54)	1.71^c^(1.38,2.13)	<0.001
Age-,gender-adjusted‡	1.0	1.11^a^(1.01,1.22)	1.15^a^(1.02,1.31)	1.38^b^(1.11,1.72)	<0.001
Multivariate adjusted§	1.0	1.08(0.97,1.19)	1.13(1.00,1.29)	1.46^b^(1.16,1.82)	0.001

*NHEFS indicates the First National Health and Nutrition Examination Survey Epidemiologic Follow-up Study; HR, hazard ratio; and CI, confidence interval.

† Number of subjects at risk for all-cause mortality (number of deaths).

**stratified by birth cohort and adjusted by age.

‡ Stratified by birth cohort and adjusted by age, gender.

§ Stratified by birth cohort and adjusted for age, gender, physical activity, level of education, regular alcohol consumption, current cigarette smoking, body mass index, occupation, eye colors, family income, vitamin supplement use, urban-rural residence, region of residence, season of the year when the examination was taken, baseline diseases, and menopause for women.

*^a^* *P*<0.05.

*^b^* *P*<0.01.

*^c^* *P*<0.001.

**Table 3:** Cancer mortality hazard ratios according to actinic skin damage and its components in 6175 NHEFS participants*.

	Actinic skin damage and its components				*P* for trend
	Absent	Minimal	Moderate	Severe	
**Actinic skin damage**					
Person-years	70666	26658	16066	1226	
Number of subjects†	3732(228)	1440(171)	924(240)	79(33)	
HR (95% CI)					
Age- adjusted**	1.0	1.01(0.82,1.23)	1.62^c^(1.34,1.96)	2.36^c^(1.63,3.42)	<0.001
Age-,gender-adjusted‡	1.0	0.94(0.77,1.15)	1.32^b^(1.09,1.61)	1.75^b^(1.20,2.55)	<0.001
Multivariate adjusted§	1.0	0.97(0.79,1.19)	1.35^b^(1.10,1.66)	1.78^b^(1.20,2.63)	0.001
**Fine Telangiectasia**					
Person-years	81151	19412	12337	1716	
Number of subject†	4307(319)	1053(136)	710(177)	105(40)	
HR (95% CI)					
Age- adjusted**	1.0	0.93(0.76,1.14)	1.61^c^(1.33,1.94)	2.04^c^(1.46,2.84)	<0.001
Age-,gender-adjusted‡	1.0	0.86(0.70,1.06)	1.29^a^(1.06,1.57)	1.51^a^(1.08,2.13)	0.004
Multivariate adjusted§	1.0	0.92(0.75,1.13)	1.33^b^(1.08,1.62)	1.47^a^(1.04,2.09)	0.003
**Solar Elastosis**					
Person-years	87502	14925	10611	1579	
Number of subject†	4643(332)	817(125)	613(171)	102(44)	
HR (95% CI)					
Age- adjusted**	1.0	1.07(0.87,1.31)	1.45^c^(1.19,1.75)	1.94^c^(1.40,2.67)	<0.001
Age-,gender-adjusted‡	1.0	0.99(0.81,1.23)	1.22^a^(1.00,1.48)	1.54^b^(1.11,2.13)	0.007
Multivariate adjusted§	1.0	1.05(0.84,1.30)	1.26^a^(1.03,1.55)	1.54^a^(1.09,2.16)	0.004
**Actinic Keratoses**					
Person-years	102541	8088	3505	482	
Number of subject†	5472(480)	460(106)	209(68)	34(18)	
HR (95% CI)					
Age- adjusted**	1.0	1.22(0.98,1.50)	1.66^c^(1.28,2.14)	2.35^c^(1.47,3.78)	<0.001
Age-,gender-adjusted‡	1.0	1.10(0.89,1.36)	1.36^a^(1.05,1.77)	1.72^a^(1.07,2.78)	0.003
Multivariate adjusted§	1.0	1.13(0.91,1.41)	1.24(0.94,1.63)	1.80^a^(1.08,2.98)	0.015

*NHEFS indicates First National Health and Nutrition Examination Survey Epidemiologic Follow-up Study; HR, hazard ratio; and CI, confidence interval.

† Number of subjects at risk for cancer (number of deaths).

**stratified by birth cohort and adjusted by age.

‡ Stratified by birth cohort and adjusted for age, gender.

§ Stratified by birth cohort and adjusted for age, gender, physical activity, level of education, regular alcohol consumption, current cigarette smoking, body mass index, occupation, eye colors, family income, vitamin supplement use, urban-rural residence, region of residence, season of the year when the examination was taken, baseline diseases, and menopause for women.

*^a^* *P*<0.05.

*^b^* *P*<0.01.

*^c^* *P*<0.001.

**Table 4:** Cardiovascular disease mortality hazard ratios according to actinic skin damage and its components in 7003 NHEFS participants*.

	Actinic skin damage and its components				*P* for trend
	Absent	Minimal	Moderate	Severe	
**Actinic skin damage**					
Person-years	73035	30110	18732	1748	
Number of subjects†	3934(430)	1739(470)	1194(510)	136(90)	
HR (95% CI)					
Age- adjusted**	1.0	1.15^a^(1.01,1.32)	1.43^c^(1.26,1.63)	2.16^c^(1.72,2.72)	<0.001
Age-,gender-adjusted‡	1.0	1.08(0.95,1.24)	1.17^a^(1.02,1.34)	1.64^c^(1.29,2.07)	<0.001
Multivariate adjusted§	1.0	1.09(0.95,1.25)	1.24^b^(1.07,1.42)	1.64^c^(1.29,2.10)	<0.001
**Fine Telangiectasia**					
Person-years	84792	22380	14339	2113	
Number of subject†	4630(642)	1322(405)	896(363)	155(90)	
HR (95% CI)					
Age- adjusted**	1.0	1.10(0.97,1.25)	1.38^c^(1.21,1.57)	1.86^c^(1.49,2.32)	<0.001
Age-,gender-adjusted‡	1.0	1.02(0.90,1.15)	1.10(0.96,1.25)	1.35^b^(1.08,1.70)	0.023
Multivariate adjusted§	1.0	1.01(0.88,1.15)	1.13(0.98,1.30)	1.34^a^(1.06,1.70)	0.013
**Solar Elastosis**					
Person-years	91394	17223	12790	2219	
Number of subject†	4969(658)	1020(328)	847(405)	167(109)	
HR (95% CI)					
Age- adjusted**	1.0	1.17^a^(1.02,1.34)	1.37^c^(1.21,1.56)	1.63^c^(1.33,2.00)	<0.001
Age-,gender-adjusted‡	1.0	1.12(0.98,1.28)	1.16^a^(1.02,1.32)	1.39^b^(1.13,1.71)	0.001
Multivariate adjusted§	1.0	1.13(0.98,1.29)	1.23^b^(1.08,1.42)	1.43^b^(1.15,1.78)	<0.001
**Actinic Keratoses**					
Person-years	108515	10054	4296	761	
Number of subject†	6001(1009)	653(299)	288(147)	61(45)	
HR (95% CI)					
Age- adjusted**	1.0	1.26^c^(1.11,1.44)	1.38^c^(1.16,1.65)	1.93^c^(1.43,2.60)	<0.001
Age-,gender-adjusted‡	1.0	1.13(0.99,1.29)	1.14(0.96,1.36)	1.44^a^(1.06,1.95)	0.006
Multivariate adjusted§	1.0	1.10(0.96,1.27)	1.14(0.94,1.36)	1.44^a^(1.05,1.98)	0.013

*NHEFS indicates First National Health and Nutrition Examination Survey Epidemiologic Follow-up Study; HR, hazard ratio; and CI, confidence interval.

† Number of subjects at risk for cardiovascular disease (number of deaths).

**stratified by birth cohort and adjusted by age.

‡ Stratified by birth cohort and adjusted for age, gender.

§ Stratified by birth cohort and adjusted for age, gender, physical activity, level of education, regular alcohol consumption, current cigarette smoking, body mass index, occupation, eye colors, family income, vitamin supplement use, urban-rural residence, region of residence, season of the year when the examination was taken, baseline diseases, and menopause for women.

*^a^* *P*<0.05.

*^b^* *P*<0.01.

*^c^* *P*<0.001.

The Cox regression results from the models with time-on-study as the time scale (data not shown) were consistent with the above-mentioned results. Subgroup analyses by age provided similar results. Incorporating sample design, excluding people who died from skin cancers, excluding subjects with diseases at baseline, and including pregnant women did not change the findings. Participants with a higher level of education, family income, or physical activity had lower risk for mortality in all of the three models. Risk factors for mortality included current cigarette smoking, baseline diseases, heavy alcohol consumption, male gender, and menopause in women. Significantly higher risks were found in both obese and underweight subjects except for the obese subjects in the cancer model. In the all-cause mortality model, having dark blue eyes was a protective factor. (Data not shown).

**Figure 1:**
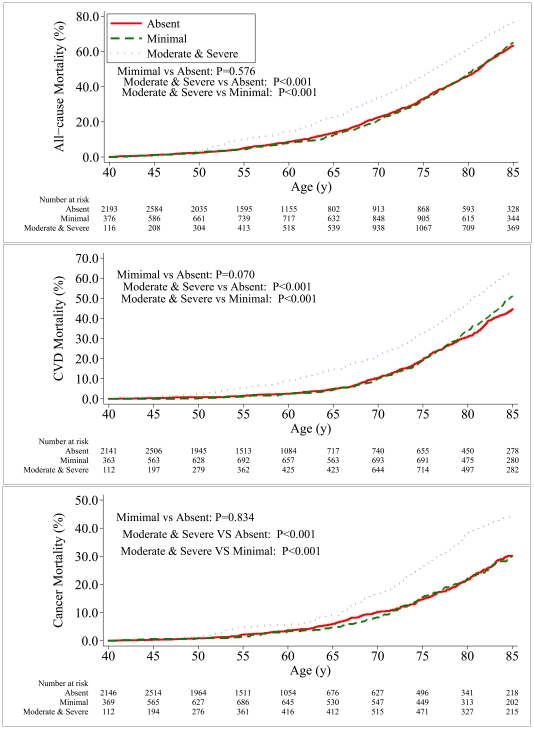
Cumulative mortality from all-cause, cardiovascular disease or cancer according to severity of overall actinic skin damage in NHEFS. Overall actinic skin damage was defined as absent, minimal, or moderate & severe. NHEFS, the First National Health and Nutrition Examination Survey Epidemiology Follow-up Study. P values were corrected by Bonferroni adjustment in multiple comparisons (p/q, q = 3, *P*<0.017).

Cumulative mortality from all causes, cancer, and cardiovascular diseases in relation to the severity of overall actinic skin damage using the Kaplan-Meier method is shown in **Figure 1**. The overall mortality curves between the minimal and no actinic skin damage groups did not differ significantly in any of the comparisons. However, participants with moderate and severe overall actinic skin damage had a significantly higher cumulative mortality rate than those with no actinic skin damage in all of the comparisons.

## Discussion

The present study in participants from white populations indicated that the risk of death from cancer, cardiovascular disease and all causes were higher for subjects with moderate to severe actinic skin damage than those with no skin damage. Subgroup analyses by age, sensitivity analyses by excluding subjects with diseases at baseline, or incorporating complex survey design did not change our results.

### Potential mechanisms

There are several possible mechanisms by which actinic skin damage may influence all-cause and cause-specific mortality. The free radical theory states that the appropriate and inappropriate production of oxidants is intricately connected to aging and lifespan. [30], [31] Many environmental stimuli, including ultraviolet (UV) radiation, can generate high levels of reactive oxygen species (ROS). [30] Plasma antioxidants are decreased in people with actinic keratosis, probably due to their long exposure to UV radiation. [32] ROS plays a role in the pathology of sun-induced skin damage, [33], [34] and thus may potentially be involved in mechanisms responsible for an increased risk of mortality in people with moderate to severe actinic skin damage reported in this study. Recent experimental and clinical data support the hypothesis that cigarette smoke exposure increases oxidative stress as a potential mechanism for initiating cardiovascular dysfunction, [35] and histologic analysis of “smoker's skin” reveals elastic fiber thickening and fragmentation, similar to that found in sun damaged skin. [36] Thus smoking and sun-related actinic skin damage may share some common mechanisms resulting in cardiovascular disease. Furthermore, repeated UV radiation can cause DNA damage and apoptosis on human peripheral blood mononuclear cells, which indicate that sunlight exposure may cause internal diseases by affecting blood components. [37], [38] In addition, UV radiation is known to activate multiple signaling cascades, leading to inflammatory reactions. [39] Despite all of these possible pathways, the mechanisms of the association between actinic skin damage and mortality remain unclear. Further study is needed to elucidate this association.

### Study implications

Previous studies have found that optimal vitamin D status may not only be important for bone health but also may protect people from some cancers and other chronic diseases. [3] It has been estimated that approximately one billion people worldwide have vitamin D deficiency or insufficiency.[3] In the United States, at the end of the winter, 42% of 15-49 year-old black girls and women had a 25-hydroxyvitamin D level below 20 ng/mL. [3], [40] Exposure to sunlight is the main source of vitamin D for most people. [41] For all of these reasons, although the American Academy of Dermatology (AAD) recommends that an adequate amount of vitamin D should be obtained from a healthy diet and/or vitamin D supplement,[42] people are encouraged to increase their sun exposure to prevent vitamin D deficiency by many studies, [3], [43], [44] and the WHO is now addressing the issue of whether current sun protection messages are too strong. [45] However, the prevalence of hypovitaminosis D was only 4.2% for white women of reproductive age, [40]. Hence to better understand the risk and benefit of sun exposure to human health is needed. It is well known that over exposure to sun increase risk for skin cancer and skin damage. If the finding from our research about the positive association between skin damage and mortality can be confirmed by other studies, an evaluation on the sun protective message should be warranted.

Children are at high-risk for sun-induced skin damage. An estimated 50% to 80% of the skin's lifetime sun damage is thought to occur in childhood and adolescence. [46] Among youths aged 11 to 18 years in the United States, 72% reported having had at least one summer sunburn, 30% reported at least 3, and 12% reported at least 5 sunburns. [47] Sunbathers are another group at high risk for actinic skin damage. [47] In a national survey, 59% of white adults reported sunbathing during the past year, and 25% reported frequent sunbathing. Of the subsample who reported sunbathing during the month before the interview, only 47% routinely used sunscreen. [48] Other high risk populations include tourists, outdoor workers, and fair skinned people living in lower latitudes. [4]

Skin damage caused by over exposure to sunlight is preventable. By running programs such as education, provision of shade in open spaces, rescheduling of work and sports activities, removal of sales taxes from approved sunscreens, and provision of cheap sunscreens, there have been substantial changes in knowledge, attitudes, and beliefs about sunlight and suntans, as well as an increase in sun protection behavior in Australia. [49]

Sunlight exposure plays an important role in both vitamin D synthesis and actinic skin damage pathology, which means that whether increased sun exposure is a protective or risk factor for chronic disease may depend on the balance between the UV induced production of vitamin D and UV induced skin damage. Throughout the United States, the estimated daily solar exposure to maintain a serum 25(OH)D level of 30 ng/mL is 15 minutes in early fall or later spring, from 11:00 AM to 2:00 PM under clear skies, assuming exposure of arms, shoulders, and back. [50], [51] Ultraviolet exposure beyond the minimal erythemal dose does not increase vitamin D production further. [51] The concentration of previtamin D in the skin reaches an equilibrium in white skin within 20 min of ultraviolet exposure. [51] However, the optimal amount of sunlight exposure a person should receive is not known but is likely to depend on many factors related to skin type, latitude, and sunscreen application. [4]

### Study limitations

A major limitation of this study is that the measurements of actinic skin damage were subject to errors of classification. [18], [52] Although the 101 examiners received training, the written instructions for this component of the examination were limited, and there were no diagnostic criteria employed in the definition of actinic skin damage severity. [52] In addition, the actinic skin damage severity evaluation was done subjectively by dermatologists. [18] However, the impact of any particular dermatologist's judgments on the overall results was minimized by the large number of examiners used in the survey. [18] In addition, the follow-up survey did not specifically collect dermatologic data [17] and we thus had to assume that the subjects' actinic skin damage did not vary over time, a factor that might result in biased hazard ratios. Another study limitation is the lack of information in potentially important characteristics such as skin type, outdoor/indoor workers or sun seeking behavior. In the present study, we used other surrogates, such as eye color, occupation and family income, to minimize these affects. Furthermore, baseline actinic skin damage data were collected almost 40 years ago, hence the study findings may not be generalizable to the current US population.

Despite these limitations, our study used a large population-based cohort with an average of 16 years of follow-up, focusing on whites only. The fact that follow-up information was available for more than 97% of the study participants and that an extensive number of covariates were available for the NHANES I subjects further enhanced the validity of our findings. Moreover, to the best our knowledge, this is the first study to investigate the relation between sun-induced skin damage and mortality.

Age could be the most important confounder in this study, because age has a very strong relationship with both actinic skin damage and all-cause mortality. [20] To adjust adequately for chronological age, we used age as the time scale and all analyses were stratified by birth cohort to control for calendar period and cohort effector. [26] Furthermore, subgroup analyses by age provided similar results. After further controlling for potential confounding factors such as smoking, occupation, region of residency, season and social-economic status, the association of over-all actinic skin damage and its components with all-cause mortality persisted, and the results remained similar when we used cancer and cardiovascular disease as the outcome.

### Conclusions

In conclusion, our study detected an association of actinic skin damage with cardiovascular disease, cancer and all-cause mortality in whites. However, this result should be interpreted with caution since unmeasured or incompletely measured confounding factors cannot be ruled out in this study and since mechanisms for these associations are lacking. More independent studies are needed before any practical recommendations can be made.
